# The Physical Diagnosis of Diseases of the Lungs

**Published:** 1843-07-22

**Authors:** 


*The Physical Diagnosis of Diseases of the Lungs.* By Walter Hayle Walshe, M. D., Professor of Pathological Anatomy in the University College, London; Physician to the Hospital for Consumption and Diseases of the Chest; Member of the Medical Society of Observation of Paris, &c. Philadelphia: Lea & Blanchard; 1843. royal 18mo. pp. 310.

This is a most admirable work. The whole subject of the physical diagnosis, as applied to the diseases of the respiratory organs, is ably and clearly treated of. It is divided into three parts. In the first part we have a general description of the methods of physical diagnosis. The different sections treat of the various means, viz.: by inspection, application of the hand, mensuration, percussion, auscultation, succession, and determination of surrounding parts and organs. The second part consists of a table exhibiting the physical cause and ordinary seat of the different physical signs, together with the names of the diseases in which they are observed, with a synopsis of signs attending each affection of the lungs, larynx and pleura. The third part is a commentary upon the two preceding ones.

"By excluding from the descriptive portion any discussion upon debateable points, and in the tabular views by distinguishing with a different type the more striking phenomena from those less constantly useful and available in practice, I have, I trust, succeeded in adapting the book for beginners. The attention bestowed upon them in the commentary will, however, show that I do not undervalue delicate signs; far from this; I know, by experience, that in many instances, apparently trivial phenomena will justify a diagnosis that, without their aid, had been altogether unwarrantable." (Preface, p. viii.)

The purely practical character of the book has not been impaired by any disquisition on acoustics, or other similar digressions. All that is said is to the point, and is said well. This is the first elementary work in which all the methods of physical investigation receive their due share of attention; all others usually being absorbed in what the author not inaptly terms an "overweening attachment to auscultation."

In conclusion, we repeat what we said at the commencement of this article—that this is a most admirable work, complete in all respects, and should be in the hands of every student of medicine, and every young practitioner.

